# Impact of Nurse Manager Leadership Styles on Work Engagement: A Systematic Literature Review

**DOI:** 10.1155/2023/5090276

**Published:** 2023-08-16

**Authors:** Amal Alluhaybi, Amanda Wilson, Kim Usher, Joanne Durkin

**Affiliations:** ^1^School of Nursing and Midwifery, Faculty of Health, The University of Technology Sydney, Sydney, NSW, Australia; ^2^School of Health, University of New England, Armidale, NSW, Australia

## Abstract

**Aims:**

This systematic review aimed to identify, evaluate, and synthesise the results of the studies that examine the relationship between nurse managers' leadership practices and staff nurses' work engagement in hospital settings and to provide recommendations for improvement and further research.

**Background:**

A lack of supportive leadership is identified as one of the most common reasons nurses leave employment. To meet the global shortage of nurses, nurse managers need to maximise staff retention and work engagement. *Evaluation*. A systematic review was conducted to identify research published between 2010 and 2021 and registered in PubMed, CINAHL, PsycINFO, Embase, EMCare, and Eric databases. The methodology guidelines outlined in the Joanna Briggs Institute (JBI) Methodology for Systematic Reviews were followed, and the results were reported using the PRISMA 2020 guidelines. The review protocol was registered with PROSPERO (ID CRD42021277463). *Key Issue*. Eleven (11) studies from 12 articles were included in this review. Three main leadership style themes were identified, and these showed statistically significant direct and indirect relationships with nurses' work engagement: relationally focused, task-focused, and lack of leadership. Work engagement was mainly assessed in terms of dedication, absorption, and vigour. The effects of leadership styles on work engagement were found to be mediated by trust in the leader, environmental resources such as structural empowerment, six work-life areas (workload, control, values, community, rewards, and fairness), person-job fit, organisational support, leader-member exchange, and personal resources such as self-efficacy and decision authority.

**Conclusion:**

This review found a significant correlation between positive nurse manager leadership style and the work engagement of registered nurses. *Implications for Nursing Management*. The results of this review suggest that nursing work engagement can be improved by implementing relational leadership behaviours. The findings of this review will be useful for developing appropriate nurse leaders' leadership styles, improving their workplace environments, and planning leadership training. It is essential to acknowledge the indirect effects of nurse leaders' leadership styles and their mediating factors on work engagement while developing interventions for staff nurses.

## 1. Introduction

Nurses account for 59% of the global healthcare workforce (World Health Organization [1]). However, there is now a shortage of around six million nurses worldwide [1], significantly affecting global healthcare systems' overall functioning [2]. Nurse shortages also result in increased burden and stress for nurses remaining in the workforce due to the greater need for overtime and poor patient/staff ratios [3–5]. These demanding work conditions may, particularly over an extended period, lead to a reduced sense of belonging and low personal satisfaction among nurses, which may result in burnout and a reduction in morale [4–6]. Feelings of disengagement and disempowerment may also lead to higher nursing staff turnover, which is again linked to poor patient outcomes and compounds existing shortages [7]. Poor patient outcomes linked to disengagement include increased incidence rates of nosocomial infections [8], rehospitalisation, medication errors, and increased mortality [3, 9, 10].

Work engagement is a positive and fulfilling state of mind concerning an individual's work. It is often characterised by the employee's vigour towards, dedication to, and absorption in their work [11], where vigour refers to the desire and ability to transfer effort into work and dedication to commitment, and absorption denotes a certain concentration and preoccupation with work [11, 12]. Work engagement in nursing is linked to better patient experiences, lower absenteeism, higher performance, and higher patient satisfaction ratings [13, 14].

Higher levels of work engagement are generally observed in healthcare organisations where managers create positive environments that allow staff to feel involved with and care for their coworkers [15]. Nurse managers play an essential role in fostering satisfaction, increasing performance among nursing staff, and creating an environment that supports professional practice [16, 17]. Nurse managers are also responsible for guiding healthcare delivery to ensure that organisational goals are met and that the best possible outcomes are attained for patients and staff [18].

Leadership is essential in shaping the general work environment and employees' perceptions of their work [19–21]. Individual leadership styles are defined by leaders' behavioural patterns in encouraging others to accomplish the common goal [22, 23]. Effective leadership styles can enhance staff motivation by encouraging autonomy, building relationships, offering resources, and employing strategies to guide, mentor, and coach staff.

Although different leadership styles are generally recognised as contributing factors to work engagement, it is unclear which styles are more effective in enhancing engagement. This systematic review aims to synthesise existing empirical research on the relationships between nurse leaders' leadership styles and nurses' work engagement.

## 2. Methods

### 2.1. Aims

This systematic review aimed to identify, evaluate, and synthesise studies that examined the relationship between nurse managers “leadership practices and staff nurse” work engagement in hospital settings and to provide recommendations for improvement of practice and further research.

### 2.2. Design

This systematic literature review was conducted in accordance with the methodology guidelines outlined in the Joanna Briggs Institute (JBI) Methodology for Systematic Reviews [24]. It was reported following the PRISMA 2020 guidelines [25]. The review protocol was registered with PROSPERO (ID CRD42021277463).

The guiding question was formulated using the PEO (population, exposure, and outcome) format, with “P” representing the staff nurses, “E,” the nurse manager's different leadership styles, and “O,” “the outcome regarding staff nurses” work engagement.

### 2.3. Search Strategy

A literature search was conducted in September 2021 using the Cochrane Library, JBI Database of Systematic Reviews, and PROSPERO to identify any previously published reviews on the relationship between nurse leadership and nursing staff nurses' work engagement. No published or ongoing reviews were found. A three-step search strategy was employed: (1) an initial, limited search using the EBSCO and OVID hosts, followed by an analysis of words from titles and abstracts and indexed terms used to describe articles; (2) a search of all identified keywords and index terms in all databases (PsycINFO, CINAHL PubMed, Embase, EMCare, and Eric); and (3) a search for additional studies in the reference lists of all identified reports and articles.

The keywords used for the search across all databases were as follows: “leadership styles OR leadership OR transformational leadership OR authentic leadership OR servant leadership OR humble leadership OR visionary leadership OR relational leadership OR resonant leadership OR supportive leadership OR transactional leadership OR laissez-faire leadership” AND “nurse manager OR nurse administrator OR supervisor ^*∗*^ OR nurse supervisor OR nurse leader OR leader ^*∗*^ OR head nurse” AND “Work engagement OR engagement ^*∗*^ OR work involvement” AND “Registered nurse OR staff nurse OR (nurse or nurses or nursing).”

#### 2.3.1. Inclusion and Exclusion Criteria

Original empirical (quantitative, qualitative, and mixed-method) research examining leadership styles of nurses in management positions and registered nurses' work engagement published in peer-reviewed journals between 2010 and 2021 was included. Studies addressing leadership development programmes, leadership instruments, leadership practices, or work engagement in professions other than nursing that did not report nursing data separately were excluded from the review. In addition, discursive papers, opinion papers, or editorials were excluded.

### 2.4. Study Selection and Outcome

**Figure 1:**
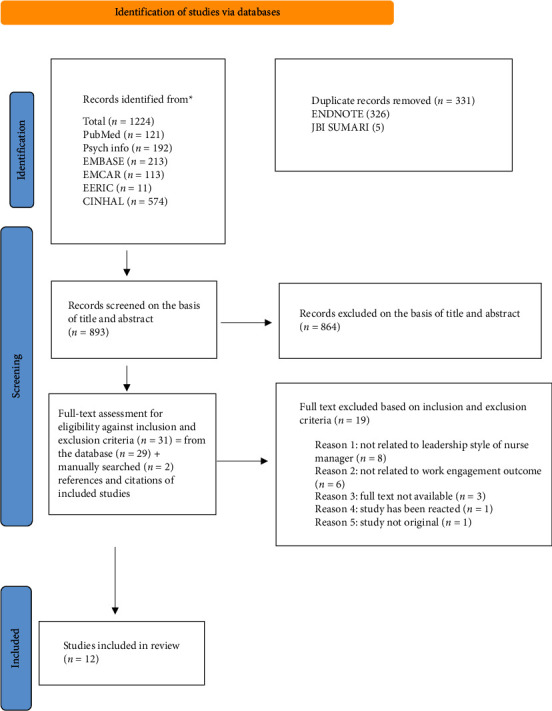
PRISMA flow chart 2020.

A total of 1224 papers were identified from the database searches, and two additional manuscripts were identified at the manual search stage. All citations were imported into the citation management tool EndNote X9 (2020), and 331 duplicates were identified and removed. The abstracts and titles were then screened independently by AM and JD using JBI SUMARI. The second reviewer, JD, checked 26% of the titles and abstracts. Then, AM and JD independently screened 31 full-text studies based on the eligibility criteria for inclusion. Of the 31 articles, 19 were excluded during the full-text screening because they did not meet the inclusion criteria. AM and JD met on three occasions to discuss and resolve any conflicts. A total of 12 articles were included in the final review. The selection process is outlined in the PRISMA flowchart shown in Figure 1.

### 2.5. Quality Appraisal

Two independent reviewers (AM and AW) evaluated the methodological quality of the 12 articles using the two-strand JBI appraisal tools. The first tool was an analytical cross-sectional for quantitative studies, and the second tool was the qualitative JBI checklist for qualitative studies. The JBI Critical Appraisal Checklist for Analytical Cross-Sectional Studies is a validated and widely used tool for appraising the quality of cross-sectional research [26]. The tool includes eight research components and a rating scale with the following response options: “yes,” “no,” “unsure,” and “not applicable” or assessing quality standards [26]. A qualitative JBI checklist assesses the congruence among the stated philosophical perspectives, methodology, objectives, data representation methods, and analysis. In addition to assessing the researcher's position on the participants and vice versa, the JBI checklist determines whether the participants' voices were represented, ensuring that ethical approvals were observed during the study. It also determines how the interpretation and results were analysed. The tools include ten research components and a rating scale with “yes,” “no,” “unsure,” and “not applicable” response options for assessing the quality standards. Reviewer disagreements regarding the bias risk were resolved three times through face-to-face and Zoom meetings.

Study quality was assessed for each study included in the review. Answers indicating “yes” scored 1 point, while answers indicating “no” or an unclear answer scored 0 points. “The Not applicable” answer was not counted. The quality rating was determined based on the sum of the points scored by each study and the total points each could earn, the quality rating was determined. These surveys were categorised as excellent (over 75%), some limitations (between 50% and 75%), and several limitations (below 50%). The articles were included in the study despite the methodological quality assessment.

In addition, AM contacted nine primary authors from the included studies to ask for additional data, specifically details about the inclusion and exclusion criteria of study participants and the confounding factors identified during the study. Four of the nine authors provided more details about their study. The critical appraisal results are reported in Tables 1 and 2.

### 2.6. Data Extraction

The data were extracted from included studies using a self-developed form. One author (AM) extracted all data relevant to the review question and piloted them in consultation with the other authors (JD, AW, and KU). One author (AM) completed the extraction with ongoing consultation with the other authors. The extracted data and study characteristics are included in Appendix 1, which can be found in the supplementary file (available here).

### 2.7. Data Analysis and Synthesis

A meta-analysis was not possible due to the heterogeneity of the instruments used to assess leadership styles and work engagement and study research design. Instead, a decision was made to use the appropriate items from the Synthesis without Meta-analysis (SWiM) [37]. The SWiM items enable studies to be grouped and guide the reporting of the standardised metrics for synthesising the study findings. Specifically, we

1. summarised each study's characteristics, results, and methodological quality
2. identified which studies were similar enough to be grouped for comparison with other study groups
3. determined which data were available for synthesis, and
4. synthesised the characteristics of the studies

## 3. Results

### 3.1. Key Characteristics of the Included Studies

This review included 12 articles comprised of 11 individual studies, of which ten used quantitative and one qualitative methodology. Two articles were considered as one study as secondary analysis [27] was published based on one article [34]. All studies included in this review were published between 2010 and 2021. Most studies (8/11) were conducted in Western countries (the United States, Ireland, New Zealand, Spain, Portugal, Canada, and Finland), while the remaining studies were conducted in Taiwan [32], Nigeria [28], and Iran [35]. The number of participants ranged from 13 to 3466 nurses. Most studies (9/11) were conducted in multiple sites, whereas two were conducted at only one site [27, 32, 34]. The participants' demographics (age, gender, educational level, and years of experience) were described in all studies with mostly female participants (97–100%), accurately reflecting gender bias in the nursing workforce globally.

### 3.2. Results of the Quality Appraisal

The quality of the included studies ranged from moderate to low. The major methodological weaknesses of the ten quantitative studies were linked to sampling methods and study design. All the quantitative studies used nonexperimental, cross-sectional data, which limited the causal findings. Most studies (7/10) did not provide details of their inclusion and exclusion criteria for study participants and/or the settings (unit context) from which the participants were recruited (8/10). All investigations used valid and reliable instruments that were criteria or psychometrically validated.

Only one study [36] used qualitative research methods. The quality of the study was rated as moderate because the article did not clearly articulate the participants' voices, but sample quotes were included as appendices. The researchers' cultural and theoretical positions in the study influence on the research and ethical considerations which were not addressed.

### 3.3. Methodology and Measurement Scales

Ten studies used quantitative methodologies with a cross-sectional design and self-reported surveys for data collection. A wide range of valid tools was used to measure leadership, including the multifactor leadership questionnaire (*n* = 5), transformational leadership behaviour inventory (*n* = 1), authentic leadership questionnaire (*n* = 1), global transformational leadership scale (*n* = 1), resonant leadership questionnaire (*n* = 1), and ethical leadership questionnaire (*n* = 1). All these studies measured work engagement using a version of the Utrecht Work Engagement Scale developed by Hu et al. [6]. The scale has three items for each of the three underlying dimensions of work engagement: vigour, dedication, and absorption. The anchors on the scale ranged from 0 (never) to 6 (always). The qualitative study by Blok et al. [36] used individual, semistructured interviews for data collection and framework analysis.

### 3.4. Leadership Styles and Nurse Work Engagement

Eight leadership styles were identified and analysed: transformational, ethical, authentic, resonant, servant, transactional, laissez-faire, and passive avoidant.

The transformational leadership style positively correlated with work engagement in 7 out of 11 cases. The authentic, ethical, resonant, and servant styles also demonstrated a 1 : 1 positive correlation. Conversely, passive-avoidant and laissez-faire styles negatively correlated with work engagement in all cases.

#### 3.4.1. Relationally Focused Leadership Styles and Nurse Work Engagement

Five leadership models, namely, transformational, authentic, resonate, ethical, and servant leadership, were analysed and described in terms of positive relational leadership behaviour and their link to improved work engagement (dedication, absorption, and vigour) in the nursing workforce. Most studies (7/11) evaluated transformational leadership [28–30, 32, 33, 35, 38], while others focused on authentic [27, 34], resonate [31], and ethical leadership [4]. Blok et al. [36] compared units with high staff engagement with those with low staff engagement in nurse leadership practice. They found that leaders' adherence to servant leadership approaches, where leaders provide help and resources to the staff to assist them in completing their work, was more frequently observed in highly engaged units. By contrast, authoritarian management styles that impose direction were more frequently found in low-engagement units [36].

#### 3.4.2. Task-Focused Leadership Styles and Nurse Work Engagement

Of the 11 studies, two showed that the transactional leadership style had a negative relationship with work engagement outcomes [29, 38]. Task-focused approaches were characterised by negotiating, supervising, and controlling; establishing goals and motivating individuals to achieve them; recognising and correcting mistakes [29, 38].

#### 3.4.3. Lack of Leadership

Two studies found that leaders with laissez-faire and passive-avoidant leadership styles negatively impacted nurse work engagement [29, 38].

### 3.5. Mediating Factor between Leadership Style and Work Engagement

A positive direct relationship between transformational leadership and staff engagement was found in two studies [29, 35]. By contrast, other studies found that leadership by nurse managers indirectly influenced work engagement outcomes through various mediating factors [4, 28, 30–33, 36, 38] specifically: staff trust in their manager [34], the six work-life areas (workload, control, values, community, rewards, and fairness) [27], structural empowerment behaviours [38], person-job fit [28], self-efficacy [33], decision authority [4], organisational support, and leader-member exchange [31].

## 4. Discussion

This review aimed to examine nurse managers' leadership styles in healthcare settings and their correlation with nurses' work engagement outcomes. Nurse managers who lead by sharing a common vision while trusting, inspiring, and advocating for their staff are perceived as more effective than those employing task-focused approaches, and a lack of clear leadership negatively impacts nurse work engagement. These findings expand on the understanding that relational-oriented leadership, which emphasises developing professional relationships with staff and maintaining high levels of interaction and trust, positively influences nursing practice outcomes. These outcomes, such as intention to stay and job satisfaction [21, 39, 40], ultimately promote quality care by improving patients' experiences and overall healthcare service satisfaction [40]. Leadership that provides a meaningful and inspiring vision motivates employees to work for a worthwhile cause despite a heavy workload [11]. Conversely, management without competent leadership (lack of accountability and poor attitude) is adverse to nurses' professional development [20, 41], leading to counterproductive work practices and facilitating factors for workplace bullying [42], which impairs patient satisfaction [43].

While effective leadership approaches had different names and frameworks, there were several common themes, including possessing ethical consideration, espousing positive behaviours, encouraging equality, and promoting healthy employee relationships. This finding is consistent with contemporary research that reveals a substantial overlap between leadership models and frameworks [38, 44–46]. The proliferation of constructs may impede organisational theory development [47]. Further research is needed to understand the impact, not just to rename models but to evaluate whether the leaders display ethical considerations, positive behaviour, and equality.

Supportive workplace environments enhance the relationship between nurse managers' leadership style and work engagement. Relational leadership contributes significantly to creating a supportive and empowering workplace. When nurses can access information and resources efficiently and have the appropriate support and autonomy to complete exemplary professional tasks, staff feel valued and more emotionally and physically connected to their work [48]. This finding builds on the understanding that a thriving and empowering working environment is critical to maximising professional nursing practice and employee turnover [3, 49–51]. Specifically, positive environments are associated with reduced adverse event reports and greater nurse-assessed quality levels, increasing the likelihood of retaining staff [3, 52–54]. However, insufficient work resources, poor communication, abusive behaviour, disrespect, and a lack of vision or leadership result in poor outcomes, such as burnout and job dissatisfaction [51]. This emphasises the significance of understanding the work context when considering both applied leadership styles and work engagement.

This review did not consider factors such as workplace culture or the nature of work that constituted the context in which leadership was exercised. When researching nursing leadership, cultural, social, and institutional contexts should be considered [19, 55, 56], failing to examine key aspects of the workplace context where patients are located, and nursing work takes place which makes it difficult to understand and analyse leadership within the nursing profession [57]. A recent realist review of healthcare leadership has shown that collaborative and transformational leadership approaches are often regarded more positively than transactional leadership practices; different practices and traits differ based on their specific contexts [58]. Furthermore, appropriate leadership behaviour depends on the circumstances and environment. Both supportively and directly, the ability to tailor leadership approaches appropriately to fit different situations is a hallmark of successful leaders [55, 59]. To develop better strategies that increase effective nurse leadership, healthcare systems should examine how context affects leadership practices and vice versa [19]. Moreover, personal and experience perspectives and expertise in context are essential, and further research should explore such contexts in greater detail. The strength of the studies included in this review is that the majority used samples from multiple sites, which increases the credibility and generalizability of findings. Weaknesses were associated with design, sampling, and low response rates. Only three of the 11 studies reported a response rate greater than 60%, limiting representativeness and introducing possible bias. There is a need for more studies using nonprobability sampling, and qualitative/mixed-method approaches to improve the overall quality of research in this field.

### 4.1. Limitations

Only peer-reviewed articles written in English were included. The samples and settings of the studies included in this review lacked heterogeneity, and most studies were conducted in Western countries, limiting the findings' generalizability. In addition, the quality of the studies included in the review ranged from moderate to low. The primary weaknesses are sampling methods and study design. Most studies featured cross-sectional, self-reported data, which may introduce response bias and limit the overall objectivity of the findings. The fact that most study participants were female might also have introduced potential gender bias; however, this reflects the inherent gender imbalance in nursing, making it less likely to be problematic. Despite these limitations, this is the first systematic review to synthesise evidence on the relationship between nursing leadership styles and registered nurse work engagement.

### 4.2. Recommendations

Future longitudinal studies are recommended to test the relationships between nurse manager leadership and staff nurses' work engagement. Various contextual and confounding factors mediating these relationships also require further examination. As most previous empirical studies that have examined nurse leadership styles and work engagement used quantitative methods, specifically cross-sectional surveys, qualitative studies focused on listening to nurses' voices to explore their experiences more deeply would be worthwhile to develop an understanding of the complexity of the phenomena investigated. Further studies in this line are recommended to further examine nurses' perceptions of work engagement.

## 5. Conclusion

There is a significant correlation between positive managers' leadership styles and the working engagement of registered nurses. Effective leadership styles share common behavioural traits that overlap with the characteristics of a positive leadership framework, such as ethical considerations, positive behaviours, equality promotion, and healthy employee relationships. The review's findings can inform the development of leadership style education and training for nursing leaders. It is important to acknowledge the indirect effects of nurse managers' leadership styles and their mediating factors when creating interventions for staff nurses.

## Figures and Tables

**Table 1:** Analytical cross-sectional study.

Citations	Q1	Q2	Q3	Q4	Q5	Q6	Q7	Q8	Total score	Share of answers yes (%)
Bamford et al. [27]	U	U	Y	Y	Y	Y	Y	U	5/8	62.5
Enwereuzor et al. [28]	Y	U	Y	Y	Y	Y	Y	Y	7/8	87.5
Garcia-Sierra and Fernandez-Castro [12]	N	N	Y	Y	Y	Y	Y	Y	6/8	75
Manning [29]	Y	U	Y	Y	Y	U	Y	Y	6/8	75
Mauno et al. [30]	N	N	Y	Y	U	U	Y	Y	4/8	50
McKenna and Jeske [4]	Y	N	Y	Y	Y	N/A	Y	Y	7/8	87.5
Parr et al. [31]	N	Y	U	U	N	N	N	U	4/8	50
Peng and Tseng [32]	N	Y	Y	Y	Y	Y	Y	Y	7/8	87.5
Salanova et al. [33]	N	N	Y	Y	N	N	Y	Y	4/8	50
Wong et al. [34]	Y	Y	Y	Y	Y	N/A	Y	Y	8/8	100
Hayati et al. [35]	N	N	Y	Y	N	N	Y	Y	4/8	50

Y = yes; N = no; U = unclear; (1) were the criteria for inclusion in the sample clearly defined?, (2) were the study subjects and the setting described in detail?, (3) was the exposure measured in a valid and reliable way?, (4) were objective, standard criteria used for measurement of the condition?, (5) were confounding factors identified?, (6) were strategies to deal with confounding factors stated?, (7) were the outcomes measured in a valid and reliable way?, (8) was appropriate statistical analysis used?.

**Table 2:** Qualitative study.

Citation	Q1	Q2	Q3	Q4	Q5	Q6	Q7	Q8	Q9	Q10	Total score	
Blok et al. [36]	U	Y	Y	Y	Y	N	N	N	Y	Y	6/10	60%

## Data Availability

Supporting data for this study are available in the article and its supplementary materials.

## Supplementary Materials

Supplementary Materials Appendix 1: Table 1 includes a study with key characteristics.
