# Supplementary material for: Impact of Nurse Manager Leadership Styles on Work Engagement: A Systematic Literature Review
Source: J Nurs Manag. 2023 Aug 16;2023:5090276. doi: 10.1155/2023/5090276 (PMC11918969; doi:10.1155/2023/5090276)
Supplement: Supplementary Materials — Appendix 1: Table 1 includes a study with key characteristics. [file 5090276.f1.docx]

**Appendix 1: Table 1 Includes a study with key characteristics.**

| **Author, year**  **and country** | **Research design and method** | **Sample** | **Response rate** | **Setting** | **Aims** | **Instrument** | **Key results** | **The mediating factor between** LS and WE |
| --- | --- | --- | --- | --- | --- | --- | --- | --- |
| Bamford et al. (2013) (Ontario) | Secondary analysis of data collected from a non-experimental, predictive design survey. | A randomised sample of 280 registered nurses | 48% RR | Acute care hospitals | To examine the relationships among nurses’ perceptions of nurse managers’ authentic leadership, nurses’ overall person–job match in the six areas of Work Life and their work engagement | - Authentic Leadership Questionnaire (ALQ) - UWE | Authentic leaders prioritize control, decision-making involvement, access to resources, recognition, teamwork, and respect, aligning with nurses' values. Nurses who work with authentic leaders report increased work engagement and more nursing experience correlates with higher work engagement. | Six areas of work life (workload, fairness, community, rewards, values) |
| Blok et al. (2021) | A qualitative approach was designed to examine perspectives using semi-structured interviews. | (*N* = 13) and managers units with high and low nurse engagement (*N* = 31) were interviewed | NA | Veteran Affairs New England Healthcare System. Leaders at five facilities | To examine nurse leaders' and managers' perspectives on employee engagement, barriers and facilitators to nurse engagement and their role in fostering engagement. | - - | Managers and leaders use different techniques to engage their staff based on their preferred management style. These styles include traditional management, servant leadership, coaching, and directive management. Traditional management involves sharing information, encouraging staff-led solutions, and offering support. Servant leaders provide team support and resources. Coaching-oriented leaders focus on individual staff development. Directive managers give clear instructions with little flexibility.  *High- versus low-engagement units*  Managers who provide resources and support to their staff have a servant leadership style, which is common in high-engagement units. Traditional management style, where managers focus on implementing directives, is more common in low-engagement units. Coaching is slightly more common in high-engagement units. Few managers follow a directive management style. |  |
| Enwereuzor et al. (2018)  (Nigeria) | Cross-sectional design | Convenience sampling of 224 nurses (15 male and 209 female) | NA | Four hospitals in Nsukka, south-eastern Nigeria | The study analysed how transformational leadership and person-job fit impact work engagement, as well as whether person-job fit moderates the relationship between transformational leadership and work engagement. | - Transformational Leadership Behaviour Inventory (TLI) - UWE | Transformational leadership positively impacted work engagement more for nurses with high Person-Job fit. No correlation was found between work engagement and demographic factors. | Person–job fit |
| Hayati et al. (2014) (Iran) | Cross-sectional design | 240 (185 women and 55 men, Mage = 25) nurses have been chosen by stratified random sampling method | NA | Five public hospitals in the Khuzestan province of Iran. | This study aimed to determine the effects of transformational leadership and its components on work engagement among hospital nurses | - MLQ - UWE | Effective leaders have the ability to inspire and motivate their team members, leading to higher levels of work engagement. They establish a clear and optimistic vision, set high expectations, and encourage employees to strive for success with passion and enthusiasm. | - |
| Manning, Jennifer (2016)  (USA) | Cross-sectional | 441 staff nurses working in 3 acute care hospitals were | NA | Three hospitals in the southeastern region of the United States. | This study aims to evaluate the influence of nurse manager leadership style factors on staff nurse work engagement. | - UWE - MLQ  5X short form | When NMs provide frequent feedback, the result is increased staff-nurse work engagement. A lack of communication and feedback from the NM decreased staff nurse work engagement. Communication is the hallmark of the transformational leadership style, and this leadership style in NMs has the potential to influence organisational success through staff nurse work engagement positively. | - |
| Mauno et al. (2016) (Finland) | Cross-sectional | A total of 3466 nurses working in the healthcare sector | 21% RR | Two healthcare labour unions (*TEHY*: the Union of Health and Social Care Professionals and *Super*: the Finnish Union of Practical Nurses) to which over 80% of Finnish nurses | To examine whether three resources, compassion, transformational leadership and work ethic feasibility, buffer against the adverse effects of emotional labour on work engagement. | - Global transformational leadership scale - UWE | Transformational leadership did not act as a buffer but showed a positive relationship with engagement. All three studied resources (work ethic feasibility, transformational leadership and compassion) were positively associated with work engagement. Transformational leadership and compassion can be valuable resources in nursing as they are positively associated with WE. |  |
| McKenna & Jeske (2021) (Ireland) | Cross-sectional | 89 nurses were recruited. | 54.7% RR | Three Irish hospitals to capture the experience of nurses between December 2017 and February 2018 | To investigate emotional exhaustion, work engagement and turnover intention in nursing by exploring the antecedent effects of ethical leadership and job components such as decision authority. | - WE - Ethical leadership | Ethical leadership style in hospitals and providing nurses with authority to make decisions can improve their work experience and help to engage, support, and retain nurses. Ethical leadership also had an indirect effect on all three outcome variables (work engagement, exhaustion, and turnover intention) | Decision authority |
| Parr et al. (2020) (New Zealand) | A cross-sectional survey designs | 252 registered nurses, enrolled nurses, and healthcare assistants, as well as administrative and clerical staff, worked in 1 of 20 units across adult inpatient medical-surgical wards | 26.4% RR | Two hospital sites in urban New Zealand. | To explore the effects of resonant leadership, leader exchange relationships and perceived organisational support on work engagement and patient outcomes | - Resonate Leadership - UWE | Strong relationships with organization leaders are crucial for better work experiences and improved patient outcomes. Resonant leadership fosters connections between staff and patients, resulting in better care quality and reduced fall rates. Focusing on engagement positively impacts staff's perception of care quality. | Organisation Support and Leader-Member Exchange |
| Peng &Tseng (2019) (Taiwan) | Cross-sectional | Two hundred thirty-four nurses and 20 supervisors, registered hospital nurses and their immediate supervisors (The., head nurses). The study sample was 100% female | NA | A regional teaching hospital of 800 beds | To explore the mediating effect of work engagement on the relationship between transformational leadership (TFL) and nurses’ job performance, along with the moderating effect of conscientiousness on work engagement within the TFL/performance relationship | - MLQ 5x-Short - UWE | TFL was positively correlated with nurses’ job performance, and the motivation mechanism of work engagement mediated the relationship between TFL and job performance, but only when nurses’ conscientiousness was high.    Employees who perceive their leaders as transformational tend to engage more at work and have better job performance. TFL is positively related to the sampled nurses’ work engagement, and work engagement’s contribution to their job performance is contingent upon their conscientiousness. | - |
| Rosa & Jordi (2018) (Spain) | Cross-sectional | A randomised sample of 131 nurses | 87.3% RR | 11 health centres with diverse levels of care (penitentiary, geriatric, mental health and primary) | To analyse the relationships among structural empowerment, the leadership style of managers and the engagement of nurses in a health organisation in Spain | - Multifactor Leadership Questionnaire (MLQ 5X short) - Utrecht work engagement (UWE) | The transformational leadership of nurse managers at the unit level has a double impact on staff nurses in the following way: first, increasing empowerment and second, increasing the engagement of the nurse staff. The transformational leadership style maintains a higher level of employment in nurses, but the transactional and the passive–avoidant styles are not predictive of engagement. Neither the control variables nor transactional or passive leadership was significant. | Leaders’ empowerment behaviour by promoting access to information, resources development opportunities and support in the workplace mediate the relationship between leader and work engagement. |
| Salanova et al. (2011) (Portuguese) | Cross-sectional design | A convenient sample of 280 nurses and their 17 supervisors | 76·9% RR | The nurses worked in different health services in a large Portuguese hospital | The link between supervisors’ transformational leadership and staff nurses’ extra-role performance as mediated by nurse self-efficacy and work engagement | - Multifactor Leadership Questionnaire (MLQ) - UWE | A direct relationship between transformational leadership and work engagement, which in turn enhances different ‘extra-role’ performances in nurses | Self-efficacy (role modelling) and verbal persuasion is a personal recourse that mediates the relationship between leadership style and WE. |
| Wong, Carol et al. (2010)  (Ontario) | Cross-sectional | A random sample of 600 registered nurses | 48% RR | Acute care teaching and community hospitals | The purpose of the present study was to test a theoretical model linking authentic leadership with staff nurses’ trust in their manager, work engagement, voice behaviour and perceived unit care quality. | - Authentic Leadership Questionnaire (ALQ) - *Work engagement* | Authentic leadership significantly and positively influenced staff nurses’ trust in their manager and work engagement (indirect), which in turn predicted voice behaviour and perceived unit care quality.  To promote trust and work engagement, it may be beneficial for managers to emphasise the four components of authentic leadership, specific behaviours such as sharing information, being open and truthful in dealing with staff, soliciting feedback from staff, involving them in decision-making and highlighting the ethical standards behind decision processes and outcomes. | Personal identification with the manager and social identification with the work.    Trust for authentic leaders and developing unconditional trust will lead to a climate where nurses are more engaged in their work and more comfortable speaking up about concerns and offering suggestions for change. |
